# Cauterization of Cancerous Degeneration of the Os Uteri by the Red Hot Iron

**Published:** 1849-02

**Authors:** 


### OBSTETRICS.

*Cauterization of Cancerous Degeneration of the Os Uteri by the Red Hot Iron.*—By M. Malgaigne and M. Jobert.—Several cases have been observed in M. Jobert's clinique, where this practice, in cases where the cancerous disease had not far advanced, was attended with the most beneficial results. The progress of the disease has been stopped, and the painful feelings of the patient have been dispelled for a time. Other cases of a similar description, and with similar results, have been lately observed among the patients of M. Malgaigne.—*Monthly Journal, from Gazette des Hôpitaux.*

